# Plasma methylated GNB4 and Riplet as a novel dual-marker panel for the detection of hepatocellular carcinoma

**DOI:** 10.1080/15592294.2023.2299044

**Published:** 2023-12-28

**Authors:** Yanteng Zhao, Lei Zhao, Huifang Jin, Ying Xie, Liyinghui Chen, Wei Zhang, Lanlan Dong, Lianglu Zhang, Yue Huang, Kangkang Wan, Qiankun Yang, Shaochi Wang

**Affiliations:** a Department of Transfusion, The First Affiliated Hospital of Zhengzhou University, Zhengzhou, Henan, China; b Plastic maxillofacial surgery, Jiangxi Provincial People’s Hospital, Nanchang, Jiangxi, China; c Translational Medicine Center, The First Affiliated Hospital of Zhengzhou University, Zhengzhou, Henan, China; d Research and development department, Wuhan Ammunition Life-tech Company, Ltd., Wuhan, Hubei, China

**Keywords:** Hepatocellular carcinoma, cell-free DNA, methylation, early stage, GNB4, Riplet

## Abstract

Early detection of hepatocellular carcinoma (HCC) can greatly improve the survival rate of patients. We aimed to develop a novel marker panel based on cell-free DNA (cfDNA) methylation for the detection of HCC. The differentially methylated CpG sites (DMCs) specific for HCC blood diagnosis were selected from The Cancer Genome Atlas (TCGA) and Gene Expression Omnibus (GEO) databases, then validated by the whole genome bisulphite sequencing (WGBS) of 12 paired HCC and paracancerous tissues. The clinical performance of the panel was evaluated using tissue samples [32 HCC, chronic liver disease (CLD), and healthy individuals] and plasma cohorts (173 HCC, 199 CLD, and 98 healthy individuals). The combination of G protein subunit beta 4 (GNB4) and Riplet had the optimal area under the curve (AUC) in seven candidates through TCGA, GEO, and WGBS analyses. In tissue validation, the GNB4 and Riplet showed an AUC of 100% with a sensitivity and specificity of 100% for detecting any-stage HCC. In plasma, it demonstrated a high sensitivity of 84.39% at 91.92% specificity, with an AUC of 92.51% for detecting any-stage HCC. The dual-marker panel had a higher sensitivity of 78.26% for stage I HCC than alpha-fetoprotein (AFP) of 47.83%, and a high sensitivity of 70.27% for detecting a single tumour (size ≤3 cm). In conclusion, we developed a novel dual-marker panel that demonstrates high accuracy in detecting HCC, surpassing the performance of AFP testing.

## GRAPHICAL ABSTRACT

**Figure:**
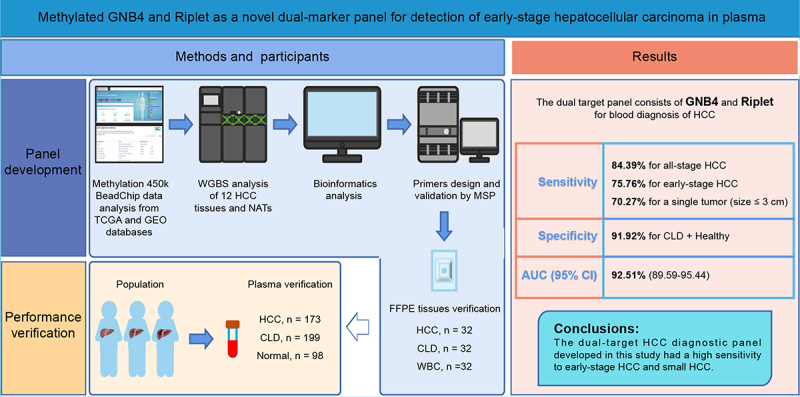

## Introduction

Hepatocellular carcinoma (HCC) is one of the most common malignant tumours and the third leading cause of cancer-related death worldwide [1]. As a high-risk country for HCC, new cases of HCC in China account for 45.3% of all new HCC cases worldwide in 2022 [2]. The high-risk population for HCC mainly includes those with hepatitis B virus (HBV) and/or hepatitis C virus (HCV) infection, excessive alcohol consumption, non-alcoholic fatty liver disease (NAFLD), cirrhosis caused by other reasons, and a family history of HCC [3,4]. Liver cirrhosis is the most critical factor in the development of HCC, with approximately 80–90% of HCC cases arising in the setting of background cirrhosis [5]. Due to the complex aetiology and heterogeneity of HCC, diagnosis of early stage HCC is difficult [6], 70–80% of patients are diagnosed in the middle and late stages of HCC [7], and the five-year survival rate is only 10.1% [8]. Early detection of HCC enables effective treatment, with the 5-year survival rates exceeding 70% for patients diagnosed at early stage [5,9]. Therefore, early detection and intervention for HCC can greatly improve the survival rate and prognosis [5,9].

Currently, ultrasound and alpha-fetoprotein (AFP) are the primary methods recommended for HCC surveillance and long-term monitoring per Chinese guidelines [10,11]. However, ultrasound has demonstrated only 47% sensitivity for early-stage HCC detection in a meta-analysis [9], and its accuracy relies heavily on operator proficiency [12]. The quality of ultrasound imaging results can also be affected by patients, such as obese patients or NAFLD patients, which can lead to an increase in false positives or indeterminate results [13,14]. While serum AFP is the most cost-effective biomarker for HCC screening, its sensitivity is suboptimal, with up to 40% of HCC patients testing AFP-negative [3]. To overcome the limitations of both ultrasound and AFP, high-performance biomarkers need to be developed.

DNA methylation is an epigenetic regulator of gene expression, which usually results in gene silencing [15]. Increased methylation of tumour suppressor genes is an early event in many tumours, suggesting that altered DNA methylation patterns could be one of the first detectable neoplastic changes associated with tumorigenesis [16–19].

Cell-free DNA (cfDNA) in the blood is a type of DNA fragment with a length of 150–200 bp released by necrotic or apoptotic normal cells or tumour cells, and those shed from tumour cells are called circulating tumour DNA (ctDNA) [20,21]. Recent research demonstrates that ctDNA can revolutionize the screening and diagnosis of cancer with a non-invasive ‘liquid biopsy’ [22–24]. The detection of ctDNA has some obvious advantages: it is minimally invasive and can reveal tumours that are not visible on imaging [22].

CtDNA containing unique cancer-associated methylation signatures has been explored as a potential biomarker for cancer detection [25]. While numerous studies have used cfDNA methylation markers for the early diagnosis of HCC [22,26], most reported cfDNA panels rely on multi-target next-generation sequencing, which is expensive and has suboptimal sensitivity for small tumours [27–29]. Therefore, it is necessary to develop an early-stage HCC detection model with fewer targets, comparable or superior diagnostic performance, and economic and convenient methods, such as methylation-specific PCR (MSP).

In this study, we identified and evaluated potential cfDNA methylation biomarkers for the diagnosis of early-stage HCC through bioinformatics analysis of The Cancer Genome Atlas (TCGA) and the Gene Expression Omnibus (GEO) database. Seven differential methylation CpG sites (DMCs) were selected and further validated by whole-genome bisulphite sequencing (WGBS) of 12 HCC tissues and matched normal adjacent tissues (NATs). GNB4 and Riplet demonstrated robust discriminative ability in the WGBS analysis and achieved the highest area under the curve (AUC) among the ROC analyses of the seven DMCs using TCGA datasets. The diagnostic performances of GNB4 and Riplet were verified in tissue and plasma samples from HCC, high-risk (cirrhosis or hepatitis), and normal cohorts. Furthermore, we compared the clinical performance of the two markers to AFP for early-stage HCC and analysed the sensitivity of the diagnosis of small HCC. Here, we present a dual-marker panel of cfDNA methylation for HCC detection using methylation-specific PCR, which can facilitate the diagnosis of small HCC tumours.

## Methods

### Study population

This study recruited a total of 217 HCC, 231 chronic liver disease (CLD), and 130 healthy participants from the First Affiliated Hospital of Zhengzhou University between June 2022 and October 2022. This clinical trial was registered at ClinicalTrials.gov (registration number NCT05685524), the American Clinical Trial Registry website, and was approved by the Ethics Committee of the First Affiliated Hospital of Zhengzhou University (2022-KY-0631-002). All data were collected in accordance with the principles of the Declaration of Helsinki and all participants signed an informed consent form.

Among the recruited participants, tumour tissues and matched NATs from 12 HCC patients were used for WGBS analysis. Tissue samples were collected from 32 HCC and CLD patients, and plasma samples were collected from 32 healthy individuals for tissue validation of the biomarkers. A total of 173 HCC patients, 199 CLD patients, and 98 healthy individuals were used for the blood validation of biomarkers.

The inclusion criteria for HCC patients were as follows: patients aged ≥18 years, clinically diagnosed with HCC, who did not receive surgery or chemoradiotherapy, and those who were excluded from other malignant tumours. The CLD group consisted of patients with chronic liver disease who were recommended for HCC surveillance but did not have a diagnosis of HCC. The CLD patients primarily recruited in this study were patients with chronic hepatitis B virus (HBV) infection, chronic hepatitis C virus (HCV) infection, and liver cirrhosis. HBV and HCV infections were determined by serological tests, and liver cirrhosis was determined by histological or imaging examinations. Healthy individuals had no clinical symptoms of liver disease or a history of cancer at enrolment.

5 ml blood were collected through the vein from all participants after enrolling them in the group. CLD tissues were collected from patients with CLD who required liver transplantation. HCC tissues and NATs were obtained during radical resection of HCC or tissue biopsy and pathologically examined. HCC patients were divided into early (stage I-II) and late (stage III-IV) stages according to the China Liver Cancer Staging System (CNLC) [10].

### DMCs selection

The HCC methylation data were obtained from TCGA database (http://cancergenome.nih.gov/), including sample types of primary tumours and normal tissues (normal, *n* = 50; tumour, *n* = 377). The β value generated by the Illumina Infinium Human Methylation 450k BeadChip was used to describe the DNA methylation levels. For CpG sites with methylation values shown as ‘NA,’ methylation values were imputed using the k-nearest neighbour algorithm (R software package ‘bnstruct’). The following criteria were used to screen for DMCs:1. The nonparametric rank-sum test was used to evaluate the significant difference between normal and tumour tissues, the *P* value was calculated, and the CpG site with *P* < 0.05 was retained; 2. β_tumour_/β_normal_ ≥2.

The GEO datasets were selected according to the following criteria: 1. Data generated using the Illumina Infinium Human Methylation 450k BeadChip; 2. Inclusion of both normal and tumour samples, with sample size exceeding 15. Three datasets (GSE89852, GSE83691, and GSE77269) underwent batch effect removal processing and were integrated into an independent dataset defined as the GEO dataset (normal, *n* = 61; tumour, *n* = 79). The DMCs were filtered according to the filtering criteria of TCGA database, and then the intersection of DMCs in the two databases was selected, as shown in Supplemental Table S1.

To eliminate interference from blood cells, the average methylation levels of intersecting DMCs were further evaluated in 656 healthy whole blood samples from the GSE40279 dataset. These were ranked from the lowest to the highest methylation levels, as lower β value indicate better performance.

High-performance plasma markers should be hepatoma-specific to discern whether cfDNA originates from hepatoma tissue or other organs/tissues. The methylation levels of the top 30 DMCs with the lowest β values in blood cells were examined across 32 cancer samples from TCGA database (the data are shown in Supplemental Table S2, Supplemental Table S3). The average β value of each DMC was calculated in each cancer type. DMCs with β values exceeding 0.3 in cancer samples were considered hypermethylated relative to cancer. Finally, seven DMCs demonstrating hypermethylation in no more than two cancer types, including HCC, were selected.

### Whole genome bisulfite sequencing and data analysis

DNA extraction and bisulphite conversion for WGBS were performed by MGI (MGI Tech Co., China). The WGBS libraries were sequenced on the DNBSEQ-T7 sequencer (MGI Tech Co., China) using paired-end sequencing (2 × 100 bp).

After base recognition, cutadapt (v 1.8.3) was used to trim all paired terminal fastq files to remove adapter sequences and low-quality bases (the quality of bases was lower than Q20, and the minimum length of reads was 36). The Hg38 (p16) reference genome was obtained from the UCSC database (https://hgdownload.soe.ucsc.edu/downloads.html). The bisulphite conversion rate was estimated by calculating the percentage of unmethylated counts for CpGs on the phage lambda genome. Reads were mapped to the reference genome using Bowtie2 mapped to t (default parameter). Then, samtools (v0.1.19) was used to sort the BAM files generated by Bismark, and the clipOverlap method of bamUtil (https://github.com/statgen/bamUtil) was applied to trim the overlapped paired-end reads to prevent duplicate counting. The methylation level of each CpG was a combination of two DNA strands, and the calculation formula was *m*/(*m*+*u*), where *m* represents the number of methylated cytosines, and *u* represents the number of unmethylated cytosines. The WGBS data from tumour and normal tissues were analysed to identify DMCs with *P* < 0.05. Differentially methylated regions (DMRs) were defined as containing at least two DMCs within 50–150 bp. Genes overlapping these DMRs were classified as differentially methylated genes (DMGs).

### ROC analysis

ROC curve analysis was performed using the R package ‘pROC’ to simultaneously estimate the AUC and 95% confidence interval (CI) of the model. The optimal sensitivity and specificity were determined at the maximum Youden index, calculated as: sensitivity + specificity − 1.

### Correlation analysis

Pearson correlation analysis was used to assess the correlation between methylation levels of DMCs shared by TCGA and WGBS data on normal and tumour samples. Spearman correlation coefficient and significance *P* value were calculated. Similarly, Pearson correlation analysis was conducted to evaluate the correlation between methylation levels and expression of target genes using TCGA-LIHC methylation and expression data. Again, the Spearman correlation coefficient and significance *P* value were determined.

### Survival analysis

Patients with HCC were divided into high and low methylation groups according to the median value of target gene methylation. Kaplan–Meier survival analysis was conducted using the Survminer and survival packages to compare the overall survival (OS) between the groups. *P* < 0.05 indicates a significant difference in OS between the high and low methylation groups.

### Extraction of DNA from tissues and plasma

Genomic DNA (gDNA) was extracted from formalin fixation and paraffin embedding (FFPE) samples of CLD and HCC tissues using the Tiangen DNA FFPE tissue kit (DP330, Tiangen Biotech (Beijing) Co., Ltd., Beijing, China) according to the manufacturer’s instructions.

After centrifuging blood collection tubes containing whole blood for 10 min (2000 rcf), the plasma was transferred to 15 ml centrifuge tubes using a pipette. The blood collection tubes containing the remaining blood cells were centrifuged again for 10 min (2000 rcf). The centrifuged plasma was combined with the plasma previously transferred to the 15 ml centrifuge tubes. The plasma was centrifuged again for 10 min (2000 rcf) and then transferred to new 5 ml centrifuge tubes. CfDNA was extracted from 2 ml of plasma using a plasma cfDNA Extraction Kit (AA16, Wuhan Ammunition, Wuhan, China). Briefly, 2 ml of plasma was added to proteinase K, lysis-binding buffer, and magnetic bead suspension, and incubated for 30 min. The magnetic bead-absorbed DNA was washed once with rinsing buffer WB1 and twice with rinsing buffer WB2. The DNA was then eluted with 50 μl TE buffer.

cfDNA quality was controlled using the Ct value of β-actin. If the Ct value was below 35, the DNA was considered valid and included in the data analysis. The cfDNA was immediately converted by bisulphite or stored at 4°C or −20°C overnight before conversion.

### Bisulfite conversion and purification

The DNA was chemically modified with sodium bisulphite to convert unmethylated cytosine into uracil, while keeping the methylated cytosine unchanged.

gDNA extracted from the tissue was converted using the DNA Conversion Kit (AA13, Wuhan Ammunition, Wuhan, China). cfDNA was converted using a Plasma cfDNA Conversion Kit (AA20, Wuhan Ammunition, Wuhan, China). Briefly, DNA was incubated with sodium bisulphite buffer and tRNA at 98°C for 10 min and at 64°C for 60 min in a PCR machine. Bisulphite-treated DNA was added to the binding buffer and the magnetic bead suspension and incubated for 15 min. The DNA was then washed once at room temperature and desulfurized for 15 min. Then, the DNA was washed twice and eluted with 50 μl TE buffer. The eluted bisulphite-converted DNA was used immediately for real-time fluorescence quantitative PCR analysis. Bisulphite-converted gDNA was named bisgDNA, while bisulphite-converted cfDNA was named biscfDNA.

### Cell lines and plasmids

A549 and HepG2 cell lines were obtained from the China Center for Type Culture Collection (CCTCC). The cells were cultured in DMEM (11960044, Thermo Fisher, MA, USA) supplemented with 10% foetal bovine serum (12484010, Thermo Fisher, MA, USA). HepG2 cells tested positive for the differential methylation region of GNB4 and Riplet genes, while A549 was a negative cell line.

The bisulphite-converted methylated sequence (Bs-M) and bisulphite-converted unmethylated sequence (Bs-UM) corresponding to the differential methylation regions of GNB4, and Riplet and ACTB genes were constructed on vector pUC57 (Wuhan GeneCreate Biological Engineering Co., Ltd. Wuhan, China). The constructed plasmids were serially diluted to 10 [3], 200, 40 copies/μl, and 8 copies/μl.

### Methylation-specific PCR and the data analysis

Methylation-specific primers and probes were verified in two ways before MSP analysis of plasma samples. The ACTB primers and probes were the same as described previously [30]. First, the amplification efficiency of the primers should be between 90.0% and 110.0%. The standard curves included 8 copies/μl to 10 [3] copies/μl of Bs-M of GNB4 plasmids for GNB4 primers and 8 copies/μl to 10 [3] copies/μl of Bs-M of Riplet plasmids for Riplet primers. Second, the primers and probes should only amplify the methylated templates tested in the methylated cell line (HepG2) and non-methylated cell line (A549) (Supplemental Figure S1, Supplemental Figure S2). The primer and probe sequences are listed in Table 1.

**Table 1.** The sequence of primers and probes.

Gene	Description	Sequence (5’-3’)
GNB4	MSP Forward Primer	CGTTATTCGGGTTTCGTTTCG
	MSP Reverse Primer	CCGAACTTCTCGCAAAAACG
	MSP Probe	AGGGGTGGTTCG
Riplet	MSP Forward Primer	TTGGGAAACGCGTTTATATTTCG
	MSP Reverse Primer	ACGAACCCTTAACTTTTTAACTCGC
	MSP Probe	AAGGCGGTAAGGATA
ACTB	MSP Forward Primer	CGCAATAAATCTAAACAAACTCC
	MSP Reverse Primer	GGGTTAGATGGGGGATATGT
	MSP Probe	TCCCAAAACCCCAACAC

For MSP to detect the bisgDNA, the volume of the PCR reaction solution was 10 μl, and 10 μl template DNA and 30 μl TE buffer were added; the total volume was 50 μl for each reaction. For MSP to detect biscfDNA, the volume of the PCR reaction solution was 10 μl and 40 μl template DNA was added. The PCR reaction solution contained: 20*HA buffer (Ammunition Life-tech, Wuhan), dNTP Mixture (Takara, Takara Biomedical Technology (Beijing) Co., Ltd.) and TaKaRa EpiTaq^TM^ HS (Takara, Takara Biomedical Technology (Beijing) Co., Ltd.). The non-template control, methylated cell line control (HepG2 cell line), was detected in each plate. PCR was carried out on an ABI 7500 instrument, and the cycling conditions were as follows: 95°C for 10 min, 95°C for 15 s, and 60°C for 30 s, 45 cycles (for bisgDNA), and 50 cycles (for biscfDNA).

After the completion of PCR, the Ct values of the target gene and internal reference gene β-actin of the sample were read, and the methylation level of each gene (2^−ΔΔCt^) in each sample was calculated. ΔΔCt = (Ct_target gene_ - Ct_internal reference_) _sample_–(Ct_target gene_ - Ct_internal reference_) _positive control_.

2^−ΔΔCt^ was used for ROC analysis, and the value of the maximum Youden index was used as the best cut-off value. The area under the ROC curve (AUC) value and the sensitivity and specificity of distinguishing cancer samples from non-cancer samples (including CLD and healthy samples) were estimated.

The calculation of sensitivities and specificities in ‘clinical performance of dual-marker panel and AFP’ was as follows:

$$\begin{array}{cc} & Sensitivity=\frac{Truepositive}{Truepositve+Falsenegative}\times 100\%\;\\ & Specificity=\frac{Truenegative}{Truenegative+Falsepositive}\times 100\%\; \end{array}$$

### Bioinformatics and statistic analysis

All bioinformatic and statistical analyses were performed using R version 3.6.1. Two groups of data were compared using the Wilcoxon rank-sum test, multiple groups of data were compared using the Kruskal–Wallis rank-sum test, and categorical variables were compared using the chi-square test. Differences were considered statistically significant at *P* < 0.05.

## Results

**Figure 1.**
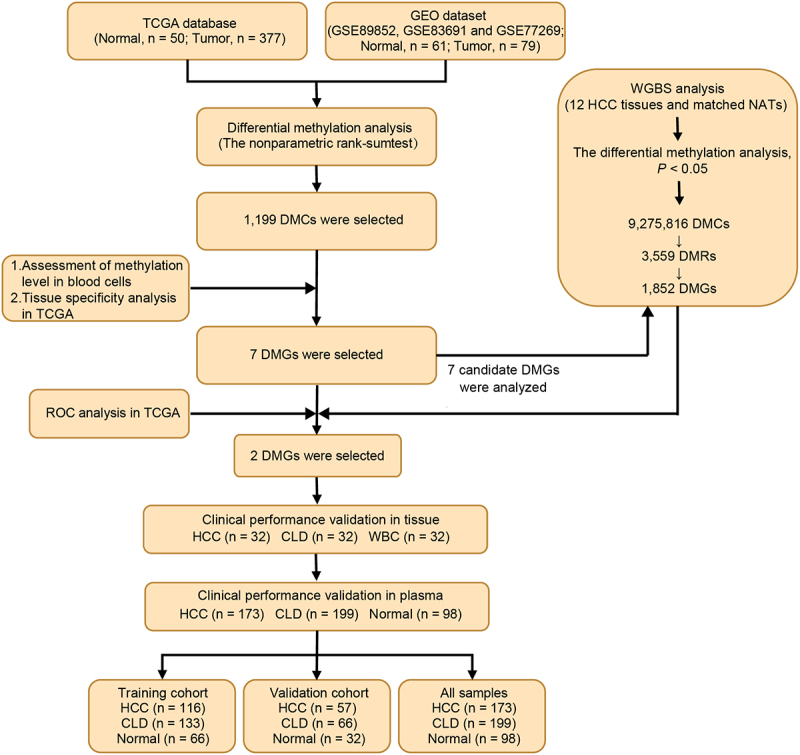
Workflow chart of identification, selection and verification of biomarkers for detection of HCC. HCC, hepatocellular carcinoma; CLD, chronic liver diseases; WBC, white blood cell; DMC, differential methylation CpG site; DMG, differential methylation gene; ROC, receiver operating characteristic.

The workflow chart of the identification, selection, and validation of novel biomarkers for the detection of early stage HCC is shown in Figure 1.

### Markers identification and selection from TCGA and GEO databases

**Figure 2.**
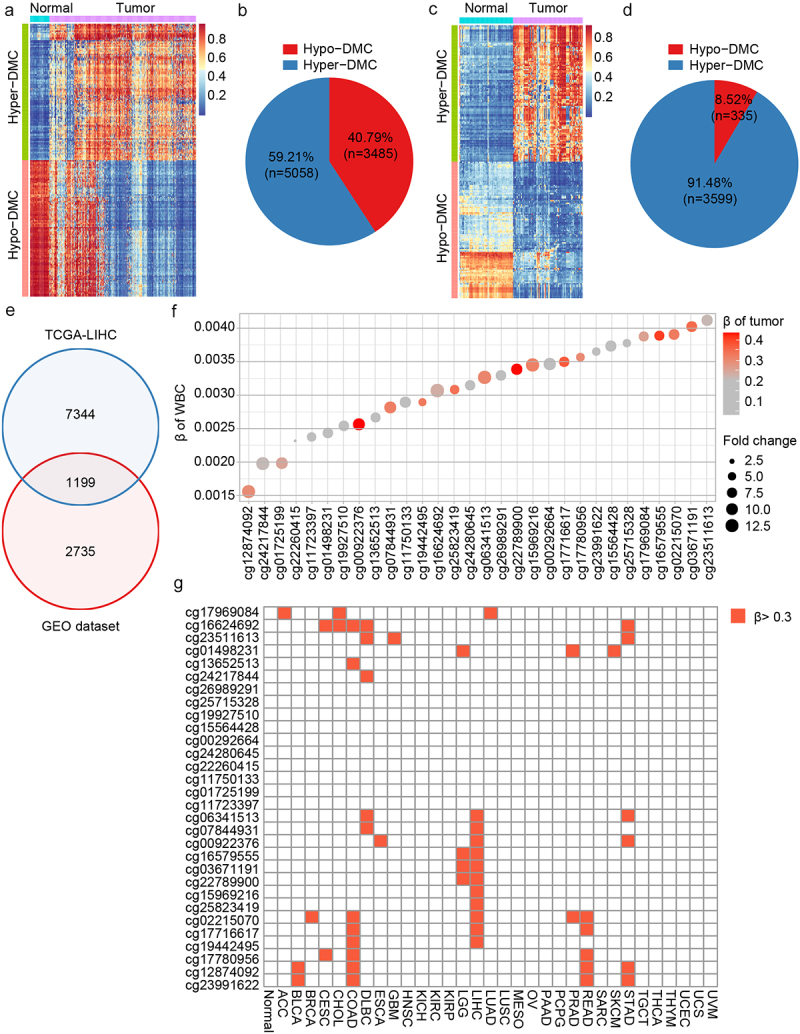
Dmcs identification and selection. a: DMCs heat map in TCGA-LIHC database (normal, *n* = 50; tumor, *n* = 377); b: In TCGA-LIHC database, pie chart of the proportion of hypermethylation DMCs and hypomethylation DMCs; c: DMCs heat map in GEO dataset (normal, *n* = 61; tumor, *n* = 79); d: In GEO dataset, pie chart of the proportion of hypermethylation DMCs and hypomethylation DMCs; e: Intersection of DMCs between TCGA-LIHC database and GEO dataset; f: The top 30 hypermethylated DMCs with the lowest β value in healthy whole blood samples. Fold change represents the ratio of methylation level of HCC to that of normal samples. The larger the circle, the greater the difference; g: Methylation levels of the 30 hypermethylated DMCs in 32 cancer types of TCGA. ACC, adrenocortical carcinoma; BLCA, bladder urothelial Carcinoma; BRCA, breast invasive carcinoma; CESC, cervical squamous cell carcinoma; CHOL, Cholangiocarcinoma; COAD, colon adenocarcinoma; DLBC, diffuse large B-cell Lymphoma; ESCA, esophageal carcinoma; GBM, glioblastoma multiforme; HNSC, head and neck squamous cell carcinoma; KICH, kidney Chromophobe; KIRC, kidney renal clear cell carcinoma; KIRP, kidney renal papillary cell carcinoma; LGG, brain lower grade Glioma; LIHC, liver hepatocellular carcinoma; LUAD, lung adenocarcinoma; LUSC, lung squamous cell carcinoma; MESO, Mesothelioma; OV, ovarian serous cystadenocarcinoma; PAAD, pancreatic adenocarcinoma; PCPG, pheochromocytoma and Paraganglioma; PRAD, prostate adenocarcinoma; READ, rectum adenocarcinoma; SARC, Sarcoma; SKCM, skin cutaneous Melanoma; STAD, stomach adenocarcinoma; TGCT, testicular germ cell Tumors; THCA, thyroid carcinoma; THYM, Thymoma; UCEC, uterine corpus endometrial Carcinoma; UCS, uterine Carcinosarcoma; UVM, Uveal melanoma.

The TCGA and GEO databases were used to screen DNA methylation markers for HCC diagnosis. Differential methylation analysis was performed on the HCC and normal groups of TCGA-LIHC methylation 450k data. This method is described in the ‘Method’ section. Analysis of the TCGA-LIHC dataset identified 8,543 differentially methylated CpGs (DMCs), with 59.21% showing hypermethylation in HCC (Figure 2a,b). An additional 3,934 DMCs were identified from the GEO database, 91.48% of which were hypermethylated in HCC (Figure 2c,d). A total of 1,199 DMCs overlapped between the two datasets (Figure 2e).

As cfDNA in blood originates from white blood cells and other tissues [31], the biomarkers used for blood diagnosis should not be interfered with by white blood cells and should be tissue-specific [32]. Thus, the methylation levels of 1,199 DMCs were evaluated in healthy white blood cells from the GEO database, and the top 30 DMCs with the lowest β value were selected (Figure 2f). To select DMCs with good HCC tissue specificity, the methylation levels of DMCs were analysed across 32 cancer types from TCGA. Seven DMCs with high HCC specificity were selected using a threshold of 0.3 (Figure 2g). They were located in the TEPP (cg07844931), Riplet (cg16579555), SPACA6 (cg03671191), MIXL1 (cg22789900), TSC22D1 (cg15969216), GNB4 (cg25823419), and CHST2 (cg19442495) genes.

### WGBS analysis and DMGs validation

**Figure 3.**
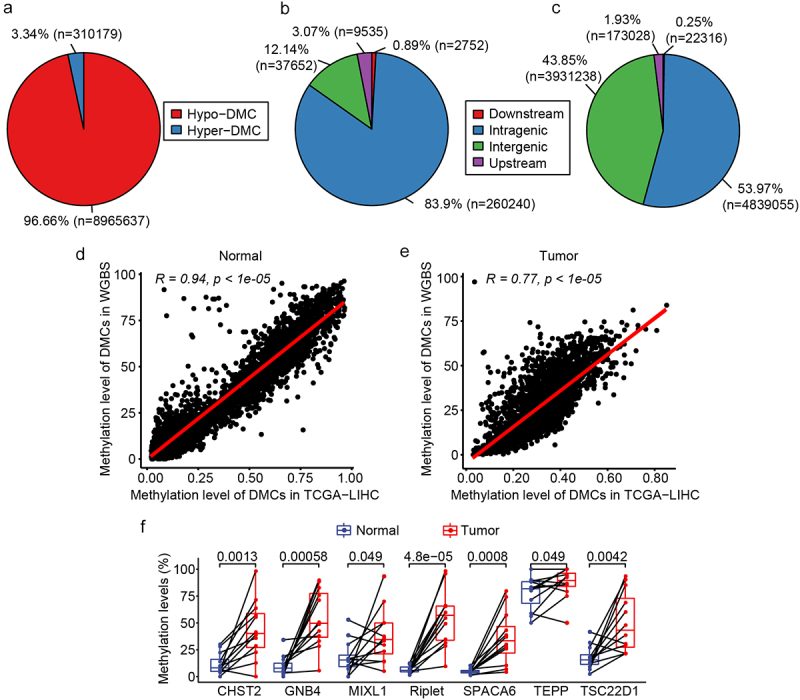
DMC and DMG analyses of WGBS. a: Pie chart of the proportion of hypermethylated and hypomethylated DMCs; b: The distribution of hypermethylated DMCs in different regions of the genome. Downstream, 0–200 bp downstream of the transcriptional start site (TSS); upstream region, 0–2000 bp upstream of the TSS; c: Distribution of hypomethylated DMCs in different regions of the genome; D: correlation of DMCs in TCGA and WGBS in normal tissues; e: Correlation of DMCs in TCGA and WGBS in tumour tissues; f: Methylation level of the seven candidate DMGs in WGBS results.

To further confirm the candidate markers for HCC diagnosis, WGBS analysis was performed on 12 HCC tissues and matched NATs. The clinical characteristics of the participants are shown in Supplemental Table S4. Of the 9,275,816 DMCs identified, 96.66% were hypomethylated in HCC compared to NATs, while only 3.34% were hypermethylated (Figure 3a). Analysis of the genomic distribution of DMCs revealed that hypermethylated DMCs were predominantly found in the intragenic region, accounting for 83.9% (Figure 3b). Hypomethylated DMCs were almost equally distributed in both the intragenic and intergenic regions, accounting for 53.97% and 43.85%, respectively (Figure 3c). In addition, 3,559 DMRs were identified based on 9,275,816 DMCs embedded in 1,852 DMGs. Comparing the DMCs analysed using TCGA database and the DMCs analysed by WGBS, it was found that the analysis results of TCGA and WGBS were highly consistent in both normal and tumour tissues (Figure 3d,e). The methylation status of the 7 candidate markers, which were selected from the analysis of TCGA and GEO databases, was further analysed in the WGBS. Of the seven genes examined, Riplet and GNB4 displayed the most significant differential methylation between HCC and adjacent normal tissues (smallest *p* values). Methylation levels of Riplet were 54.90% in HCC tissues compared to just 6.78% in adjacent normal tissues. Similarly, GNB4 methylation was 54.52% in HCC tissues versus 10.52% in adjacent normal tissues. CHST2, SPACA6, and TSC22D1 had lower methylation levels in cancer tissues than Riplet and GNB4, in which CHST2 and TSC22D1 had higher methylation levels in normal tissues (Figure 3f). There was only one DMC in MIXL1 and TEPP; thus, both were not among the 3,559 DMRs of the WGBS results. These results are shown in Figure 3f for comparison. The above results indicated that the WGBS data were consistent with TCGA data, and seven DMGs were hypermethylated in HCC compared to normal tissues, of which Riplet and GNB4 showed great discrimination ability in TCGA, GEO, and WGBS analyses.

### Dual markers selection from the analysis of TCGA database

We further evaluated the diagnostic performance of the seven candidate DMGs using ROC analysis in TCGA datasets. The results showed that the AUC values of any single DMG were smaller than those of any combination of two DMGs, indicating that the combination of two DMGs could improve the performance of an assay for HCC detection (Table 2). Of all the two-marker combinations tested (*n* = 21), the combination of Riplet and GNB4 markers had the highest AUC value (91.47%), with 100% specificity and 83.02% sensitivity. This suggested that the panel of Riplet and GNB4 could be the optimal combination, which is also consistent with TCGA, GEO, and WGBS analyses. Further investigation revealed that both Riplet and GNB4 were hypermethylated at the early stage of HCC (Supplemental Figure S3A, B). Riplet and GNB4 did not show significant variation in detecting early- and late-stages of HCC (Supplemental Figure S3A, B), indicating that they can be used to diagnose early stage HCC.

**Table 2.** ROC analysis of single gene and dual gene panels.

DMG	AUC, % (95% CI)	Sensitivity, %	Specificity, %
Riplet	84.33 (81.15–87.66)	78.51	100.00
TEPP	81.27 (76.47–85.56)	68.97	100.00
MIXL1	80.60 (77.48–84.86)	77.72	100.00
CHST2	80.56 (77.47–83.44)	74.80	96.00
TSC22D1	79.29 (75.56–84.46)	63.66	98.00
GNB4	77.18 (71.86–80.54)	67.90	100.00
SPACA6	75.74 (72.05–79.94)	67.64	98.00
Riplet+GNB4	91.47 (88.83–94.17)	83.02	100.00
SPACA6+Riplet	91.45 (88.79–93.98)	86.47	100.00
CHST2+Riplet	91.18 (88.61–93.76)	84.62	98.00
MIXL1+Riplet	91.17 (89.00–93.74)	87.80	100.00
TSC22D1+Riplet	90.69 (87.99–93.13)	84.62	100.00
TEPP+Riplet	90.11 (86.99–92.83)	85.41	100.00
CHST2+MIXL1	89.34 (86.98–91.75)	84.35	100.00
SPACA6+CHST2	88.99 (85.79–91.28)	84.35	96.00
MIXL1+TEPP	88.68 (85.62–91.58)	85.68	100.00
CHST2+GNB4	88.67 (85.63–91.47)	80.64	98.00
TEPP+TSC22D1	88.67 (85.82–91.24)	78.51	100.00
TEPP+GNB4	88.49 (85.71–90.97)	80.90	100.00
SPACA6+TEPP	87.82 (84.32–90.85)	81.96	98.00
SPACA6+GNB4	87.43 (83.83–90.81)	79.84	98.00
CHST2+TEPP	87.39 (84.47–90.28)	82.49	98.00
MIXL1+GNB4	87.33 (84.31–90.16)	80.11	100.00
SPACA6+MIXL1	87.14 (83.39–90.16)	84.08	98.00
SPACA6+TSC22D1	86.57 (82.60–88.81)	77.98	98.00
CHST2+TSC22D1	86.50 (83.54–88.98)	78.51	100.00
MIXL1+TSC22D1	85.76 (83.00–88.30)	81.17	98.00
TSC22D1+GNB4	85.46 (81.84–88.67)	74.01	100.00

**Figure 4.**
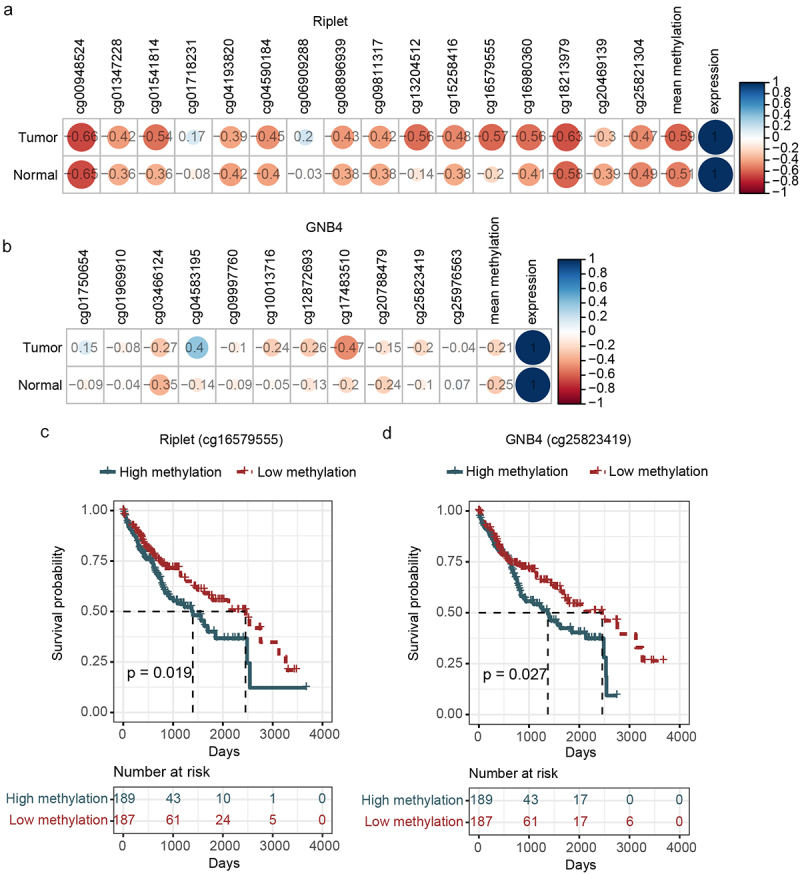
Correlation analysis of the methylation and expression, survival curve analysis of CpG sites methylation of riplet and GNB4 by using TCGA datasets. a: Correlation matrix between methylation level and expression of all CpG sites in riplet gene; b: Correlation matrix between methylation level and expression of all CpG sites in GNB4 gene. The numbers in the circles represented correlation coefficients, with red indicating negative correlation and blue indicating positive correlation; c: Overall survival curves of HCC patients with different methylation levels of the Riplet; d: Overall survival curves of HCC patients with different methylation levels of the GNB4. There was statistic significance when *p* < 0.05.

A correlation analysis using TCGA datasets was performed to study the relationship between methylation and mRNA levels. The results showed that Riplet and GNB4 methylation levels were negatively correlated with their mRNA expression levels (Figure 4a,b). Prognostic analysis also found that the DMCs for Riplet and GNB4, selected by TCGA analysis, significantly impacted the prognosis of HCC (Figure 4c,d), indicating that their methylation levels may be used for prognosis evaluation. However, Riplet and GNB4 mRNA levels did not correlate with prognosis (data not shown).

### Verification of Riplet and GNB4 in tissues

The aforementioned results indicate that methylated GNB4 and Riplet showed optimal performance in discriminating HCC from normal controls. We then verified their methylation status and clinical performance in a cohort of 32 HCC tissues, 32 CLD tissues, and 32 healthy WBC. The clinical characteristics of the participants are shown in Supplemental Table S5.

**Figure 5.**
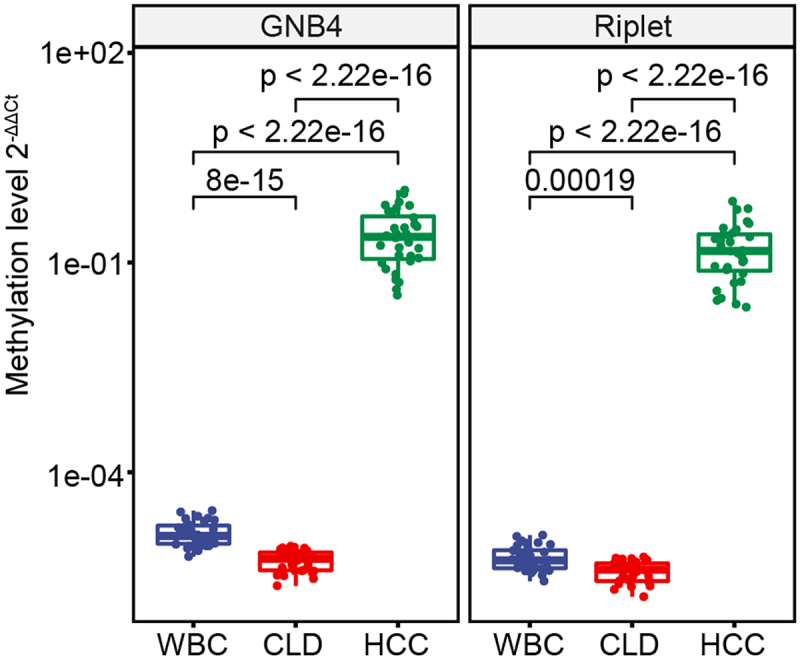
The methylation levels of GNB4 and Riplet were detected in HCC tissues, CLD tissues, and healthy WBC.

MSP analysis showed that methylation levels of GNB4 and Riplet, as measured by 2^−ΔΔCt^ values, were significantly higher in HCC tissues compared to CLD tissues and WBC, which had relatively low methylation (Figure 5). The diagnostic performance of GNB4 and Riplet methylation in detecting HCC was assessed, revealing 100% sensitivity and 100% specificity in discriminating HCC from CLD with an AUC of 100% for each gene individually and in combination (Table 3).

**Table 3.** Clinical performance of methylated GNB4 and Riplet for detecting HCC in tissue samples.

Biomarkers	Sensitivity, % (HCC, *n* = 32)	Specificity, % (CLD, *n* = 32)	AUC, % (95% CI)
GNB4	100	100	100 (100–100)
Riplet	100	100	100 (100–100)
GNB4 + Riplet	100	100	100 (100–100)

### Clinical performance of Riplet and GNB4 in plasma

It has been verified in tissue samples that methylated GNB4 and Riplet show optimal performance in discriminating HCC from CLD controls. We further verified their clinical performance in plasma cohorts consisting of 173 HCC plasma and 297 non-HCC plasma samples [199 CLD patients and 98 healthy individuals (Normal)]. The clinical characteristics of the study participants are presented in Table 4.

**Table 4.** Clinical characteristics of study cohort.

	HCC				CLD			Normal	
	Training	Validation	All samples	Training	Validation	All samples	Training	Validation	All samples
Total, n	116	57	173	133	66	199	66	32	98
Age, years									
Mean ± SD	59 ± 13	59 ± 8	59 ± 11	48 ± 13	52 ± 13	49 ± 13	40 ± 13	39 ± 10	39 ± 12
Gender									
Male	89	50	139	91	46	137	40	17	57
Female	27	7	34	42	20	62	26	15	41
Hepatitis virus infection									
Yes	85	43	128	80	34	114			
No	1	1	2	5	4	9			
UNK	30	13	43	48	28	76			
Cirrhosis									
Yes	92	49	141	102	53	155			
No	3	1	4	1	2	3			
UNK	21	7	28	30	11	41			
Child-Pugh class									
A/B	99	48	147	51	28	79			
C	7	4	11	30	15	45			
UNK	10	5	15	52	23	75			
CNLC stage									
I	44	22	66						
II	21	10	31						
III-IV	51	25	76						
Number of tumour									
1	60	36	96						
≥2	49	18	67						
UNK	7	3	10						
Singel tumour size									
≤3 cm	14	9	23						
>3 cm	41	27	68						

Note: UNK = Unknown.

**Figure 6.**
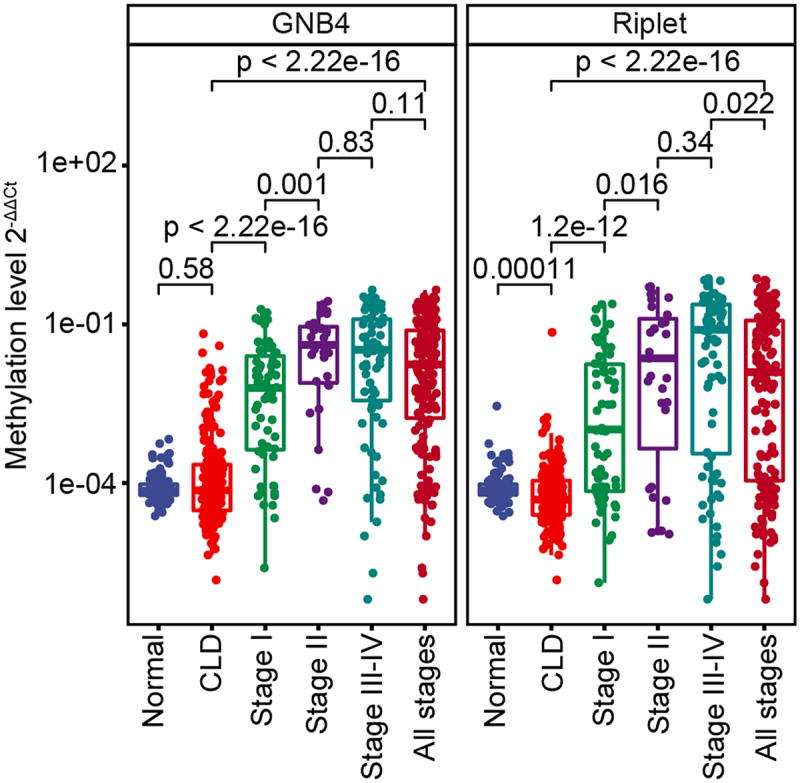
The methylation level of GNB4 and Riplet in all plasma samples.

The samples were randomly divided into training and validation cohorts in a 2:1 ratio. The methylation levels of GNB4 and Riplet in all samples determined by MSP are shown in Figure 6. ROC analysis was performed for training, validation, and all sample cohorts for HCC detection of GNB4 and Riplet. The training cohort and all samples had similar clinical performance, whereas the validation cohort showed slightly better performance.

**Figure 7.**
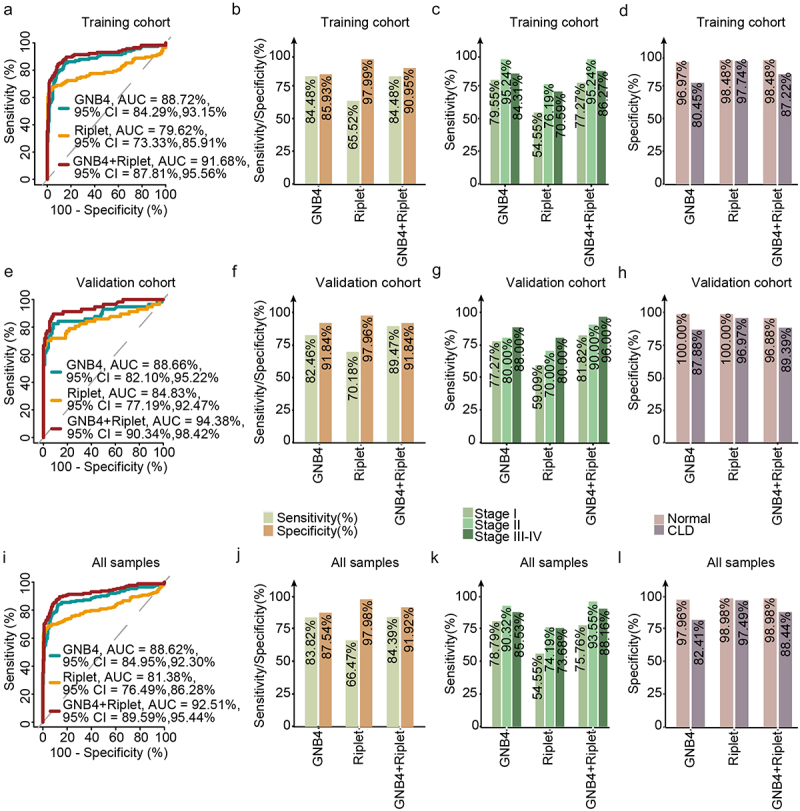
Evaluation of clinical performance of methylated GNB4 and Riplet in blood diagnosis of HCC. a, e, and i were ROC analysis of GNB4, Riplet, and GNB4+Riplet in training cohort, validation cohort and all samples, respectively; b, f, and j: The sensitivity and specificity of GNB4, Riplet, and GNB4 + Riplet in the diagnosis of HCC from non-HCC samples in the training cohort, validation cohort, and all samples were calculated based on the optimal cut-off values determined by ROC analysis; c, g, and k: The sensitivity of GNB4, Riplet, and GNB4 + Riplet to diagnose different stages of HCC in training cohort, validation cohort and all samples; d, h, and l: The specificity of GNB4, Riplet, and GNB4 + Riplet in the diagnosis of HCC in normal individuals and CLD patients in training cohort, validation cohort and all samples.

The clinical performances of the two genes and a single gene were compared. In all sample cohorts, GNB4 combined with Riplet had an AUC of 92.51% for any-stage HCC detection, which was higher than that for any single gene (GNB4:88.62%; Riplet:81.38%) (Figure 7i). GNB4 had a high sensitivity of 83.82% for any-stage HCC detection, but a relatively low specificity of 87.54% in CLD and healthy cohorts; Riplet had a relatively low sensitivity of 66.47% for any-stage HCC detection, but a high specificity of 97.98% in CLD and healthy cohorts. When the two genes were combined, the sensitivity improved to 84.39%, with a specificity of 91.92% for HCC diagnosis (Figure 7j). The trend was the same for the training and validation sets (Figure 7).

GNB4 combined with Riplet demonstrated sensitivities of 75.76% and 93.55% for stage I and stage II HCC detection, respectively, and 88.16% for stage III – VI in the entire sample cohort, indicating that the combined diagnosis of the two genes had good sensitivities in early and late HCC detection (Figure 7k). The specificity in healthy people was 98.98%, and in the CLD population was 88.44%, indicating that the combined diagnosis of the two genes had good specificity in different cohorts (Figure 7l).

In summary, the diagnostic performance of GNB4 combined with Riplet was better than that of a single-gene diagnosis. They achieved high diagnostic sensitivity in the early stage of HCC and high diagnostic specificity for different populations.

### Clinical performance of dual-marker panel and AFP

AFP is a serological indicator that is commonly used in the clinical diagnosis of HCC. We compared the clinical performance of GNB4 and Riplet and AFP using HCC and CLD samples with AFP indicators from the patients listed in Table 4.

The sensitivity and specificity of GNB4 combined with Riplet were calculated using the cut-off values of all sample cohorts (Figure 7). The early- and all-stage sensitivity of GNB4 and Riplet was consistent with that of AFP at the commonly used clinical cut-off value of 20 ng/ml (stage I: 78.26% vs. 78.26%; all stage: 86.30% vs. 86.30%), but the specificity was significantly higher (93.55% vs. 70.97%) (Table 5). To compare the sensitivity of these two methods more directly, we set the specificity of AFP to 93.55%, which is consistent with the specificity of GNB4 and Riplet, by changing the AFP threshold. AFP with a cut-off value of 209 ng/ml showed much lower sensitivities for early and all-stage HCC than GNB4 and Riplet (stage I: 47.82% vs. 78.26%; all stages: 61.64% vs. 86.30%). Through the analysis of the two detection methods, we found that GNB4 and Riplet was superior to AFP in terms of overall performance for diagnosing HCC.

**Table 5.** Clinical performance of GNB4 combined with Riplet and AFP for HCC.

	CNLC stage sensitivity (%)				
Biomarker (cut-off)	Stage I (*n* = 23)	Stage II (*n* = 11)	Stage III-IV (*n* = 39)	All stages sensitivity (%) (*n* = 73)	Specificity (%) (*n* = 31)
GNB4 + Riplet	78.26	100.00	87.18	86.30	93.55
AFP (≥ 20 ng/ml)	78.26	90.91	89.74	86.30	70.97
AFP (≥ 209 ng/ml)	47.83	54.55	71.79	61.64	93.55

**Figure 8.**
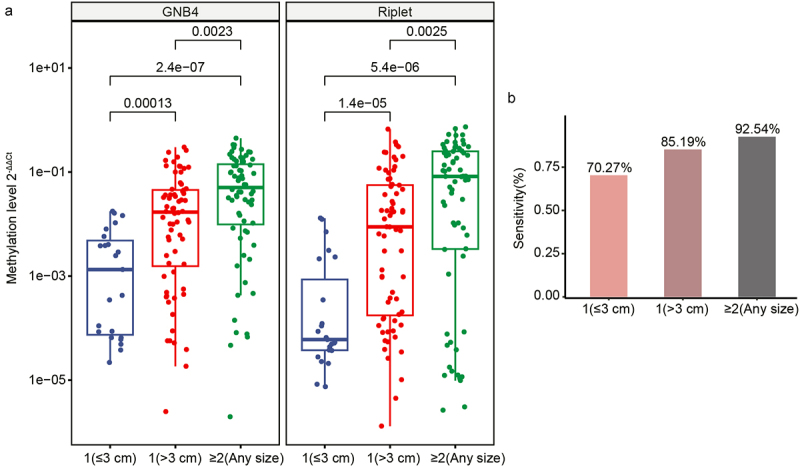
Clinical performance of GNB4 and riplet for different tumour sizes of HCC. a: The methylation level of GNB4 and riplet in different tumour sizes of HCC; b: The sensitivity of GNB4 + riplet to distinguish different tumour sizes of HCC.

The current diagnosis of HCC is limited by the challenge that small tumour volumes are difficult to identify accurately [33]. Thus, we further analysed the sensitivity of GNB4 and Riplet for the diagnosis of tumours with different diameters. The results showed that the methylation levels of GNB4 and Riplet showed a gradual increase corresponding to larger tumour size and greater tumour number. This implies that the methylation levels of these two genes are related to the size and number of tumours (Figure 8a). The sensitivity of these two genes to diagnose tumour size and number was calculated. The results showed that the sensitivity of GNB4 and Riplet was 70.27% for a single tumour with a diameter of less than 3 cm, whereas the sensitivity was as high as 92.54% for more than two tumours (Figure 8b). The results showed that GNB4 and Riplet can also assist in the clinical diagnosis of HCC with small tumours.

## Discussion

The liver has a higher blood supply than other organs, and ~ 1% of cfDNA is derived from hepatocytes [31]. Many studies have shown that the detection of plasma cfDNA methylation has strong application potential for the diagnosis of HCC [19,25,34].

In this study, the public data from TCGA and GEO databases were first used to conduct differential methylation analysis. Steps were taken to exclude interference from white blood cells and identify DMCs highly specific to HCC tissues. TCGA and GEO utilized the 450k Beadchip for DNA methylation profiling. However, whole genome bisulphite sequencing (WGBS) represents the gold standard for evaluating cytosine methylation at the single base resolution with superior accuracy [35]. Therefore, to further validate findings from TCGA and GEO, we performed WGBS on 12 pairs of tumour and adjacent normal tissues from Chinese HCC patients. Our results (Figure 3a-c) align with the known oncogenic phenotypes of methylation, which are global hypomethylation of the cancer genome and focal hypermethylation at tumour suppressor gene promoters [16]. Additionally, the seven candidate markers identified from TCGA and GEO were also hypermethylated in our WGBS data, with Riplet and GNB4 showing the greatest differences between HCC and normal (Figure 3f). Subsequent ROC analysis of TCGA data revealed the GNB4 and Riplet combination had the highest AUC among all two-marker combinations. These findings demonstrate consistency between the TCGA and our WGBS methylation data.

The clinical performance of GNB4 and Riplet in all plasma sample cohorts showed good performance, 75.76% sensitivity for stage I and the specificity was 91.92% for cohorts of healthy and CLD; the AUC was 92.51%. In other multi-target HCC diagnosis panel studies, the HCC blood screening model based on next-generation sequencing (NGS) contains 2,321 differential methylation markers. The sensitivity to HCC is similar to that of our panel (any stage:84% vs. 84.39%; early stage vs. stage I:76% vs. 75.76%), and the diagnostic specificity and accuracy of both methods are also above 90% or 0.9 (specificity: 96% vs. 91.92%; AUC: 0.957 vs. 0.925) [19]. However, the detection cost and operation time of our dual-target panel based on qPCR are significantly lower than those of the screening model based on NGS. Another multi-target HCC diagnostic panel based on PCR composed of four DNA methylation markers (HOXA1, EMX1, TSPYL5, and B3GALT6) and two protein markers (AFP and AFP-L3) had a slightly lower sensitivity (80% vs. 84.39%; early stage vs. stage I:71% vs. 75.76%) and specificity (90% vs. 91.92%) than our panel [36]. Another six-marker HCC diagnostic panel based on PCR composed of six DNA methylation markers (HOXA1, EMX1, AK055957, ECE1, PFKP, and CLEC11A, normalized by B3GALT6 level) had higher diagnostic sensitivity (any stage: 95% vs. 84.39%; early stage vs. stage I: 93% vs. 75.76%) and similar specificity (92% vs. 91.92%) compared to ours [26]. The dual-target panel in this study achieved a diagnostic performance comparable to that of the multi-target panel, with a smaller sample amount (plasma of 2 ml vs. 5 ml [37], shorter treatment time, and lower cost than that of the multi-target panel.

Recently, it has been reported that DNA methylation of G protein subunit beta 4 (GNB4) can be a potential target for anti-oestrogen resistance treatment of breast cancer and a potential marker for diagnosis and prognosis evaluation of gastric cancer induced by *Helicobacter pylori* [38,39] The Riplet gene (also known as Ring finger protein 135, RNF135) has also been found to be involved in the regulation of proliferation and metastasis of triple-negative breast cancer [40], and its promoter methylation is related to the immune invasion and prognosis of HCC [41]. These studies have shown that methylation of these two genes has an important regulatory role in the occurrence and development of cancer. We also found that these two genes showed hypermethylation at the early stage of HCC, and the methylation level was significantly negatively correlated with the transcript, indicating that GNB4 and Riplet are highly likely to influence the occurrence and development of HCC by regulating gene expression through methylation and can be used for the early diagnosis of HCC.

The GNB4 and Riplet test was validated in another study. They reported that the sensitivity of the methylated GNB4 and Riplet assay kit for HCC detection provided by Wuhan Ammunition Life-tech Company, Ltd. was 88.9%, with a specificity of 100.0% based on Ct values method on a cohort of 28 samples [42]. These findings are consistent with our results. Methylated GNB4 and Riplet have also been reported to improve the diagnostic sensitivity and specificity of circulating tumour cell (CTC) counts for HCC, increasing them from 70.6% and 90.9% to 88.2% and 100%, respectively [42].

AFP is a common blood marker for HCC detection in the clinic, although it has poor sensitivity for HCC, especially for early HCC [11]. Our dual-target HCC panel overcame this problem, and its sensitivity to HCC was significantly higher than that of AFP (86.3% vs. 61.64%), particularly for early stage HCC (78.26% vs. 47.83%). HCC with a diameter of less than 3 cm have relatively benign pathological characteristics. Good long-term survival can be achieved by surgical resection or liver transplantation for small tumours [43–45]. In this study, panel detection had a high sensitivity of 70.27% for single small tumours with diameters ≤3 cm, which can prompt HCC patients to conduct early intervention to improve their survival rate and prognosis.

This study has some obvious limitations. First, this was a single centre case-control study with regional restrictions on patients, which can easily overestimate the performance of the biomarker. Therefore, a large amount of multi-centre clinical data is required to further verify the panel’s performance. Currently, we are conducting a multi-centre clinical trial (NCT05668793) to validate the performance of dual-target panels in HCC diagnosis. Secondly, the performance of the dual-marker panel needs to be validated in other patient populations with varying aetiologies and epigenetic profiles. This includes validation in patients with different aetiological categories (HBV, HCV, alcohol, etc.) as well as populations in other countries where genetic backgrounds may differ. Finally, whether the socioeconomic effect of this method meets the requirements for clinical diagnosis in the current society needs further research.

## Conclusions

In conclusion, the dual-target HCC diagnostic panel developed in this study had a high sensitivity for early stage HCC and small HCC, which demonstrated that our panel could realize an early diagnosis and improve the survival rate and prognosis of patients. The clinical performance of GNB4 and Riplet was far superior to that of AFP, which is expected to improve the performance of the existing clinical diagnostic methods for HCC.

## Supplementary Material

Supplemental Table S4.xlsx

Supplemental Table S3.xlsx

Supplemental Table S2.xlsx

Supplemental Table S5.xlsx

Supplemental Table S1.xlsx

Supplemental Materials.docx

## Data Availability

The datasets used and/or analysed during the current study are available from the corresponding author upon reasonable request.
