# Supplementary material for: Plasma methylated GNB4 and Riplet as a novel dual-marker panel for the detection of hepatocellular carcinoma
Source: Epigenetics. 2023 Dec 28;19(1):2299044. doi: 10.1080/15592294.2023.2299044 (PMC10761049; doi:10.1080/15592294.2023.2299044)
Supplement: Supplemental Materials.docx [file KEPI_A_2299044_SM5953.docx]

**Supplemental Materials**


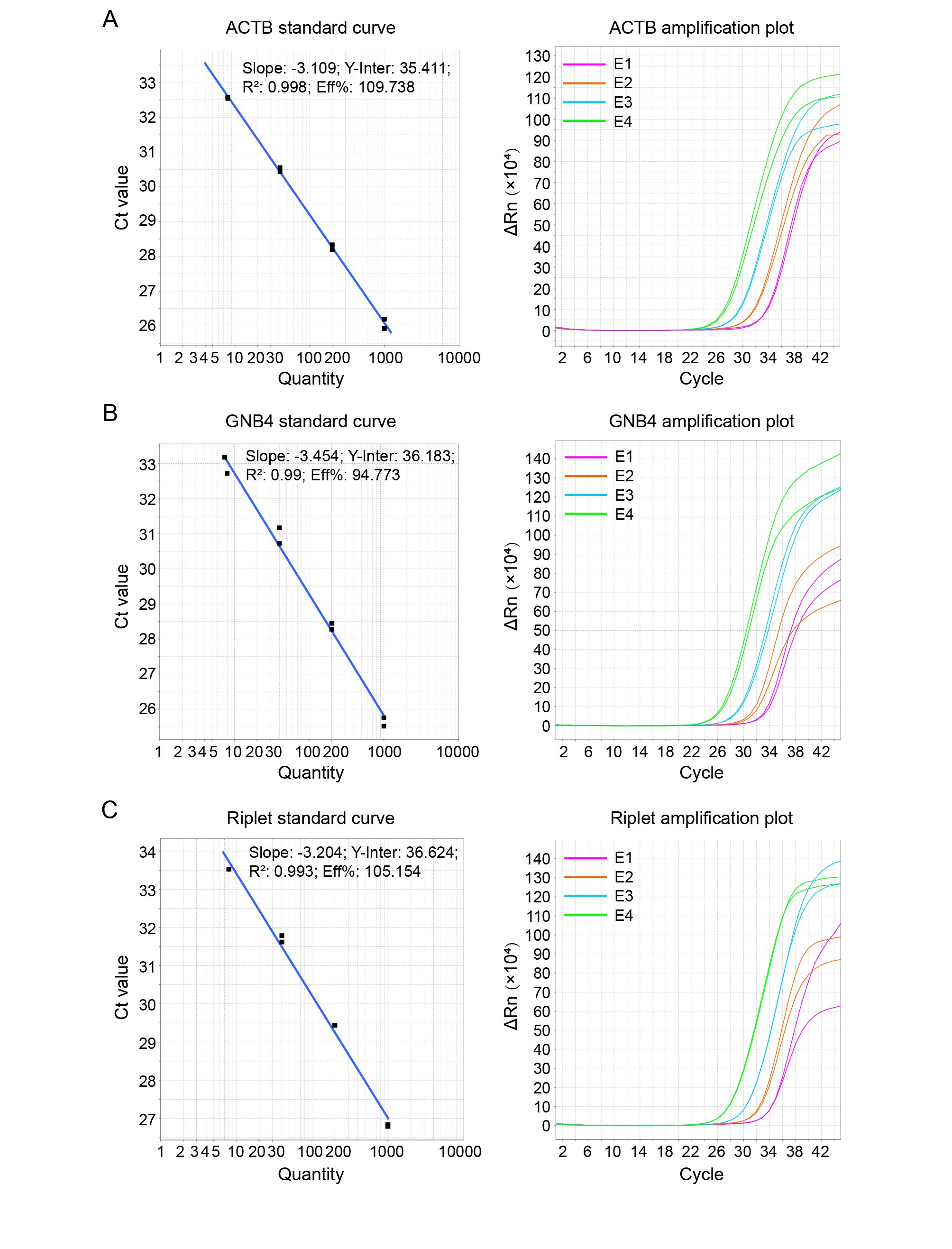


Supplemental Figure S1: The amplification curves and standard curves. A, B, and C were the amplification curves and standard curves of ACTB, GNB4, and Riplet primers for different template concentrations. E1: 8 copies/μl, E2: 40 copies/μl, E3: 200 copies/μl, E4: 1000 copies/μl.


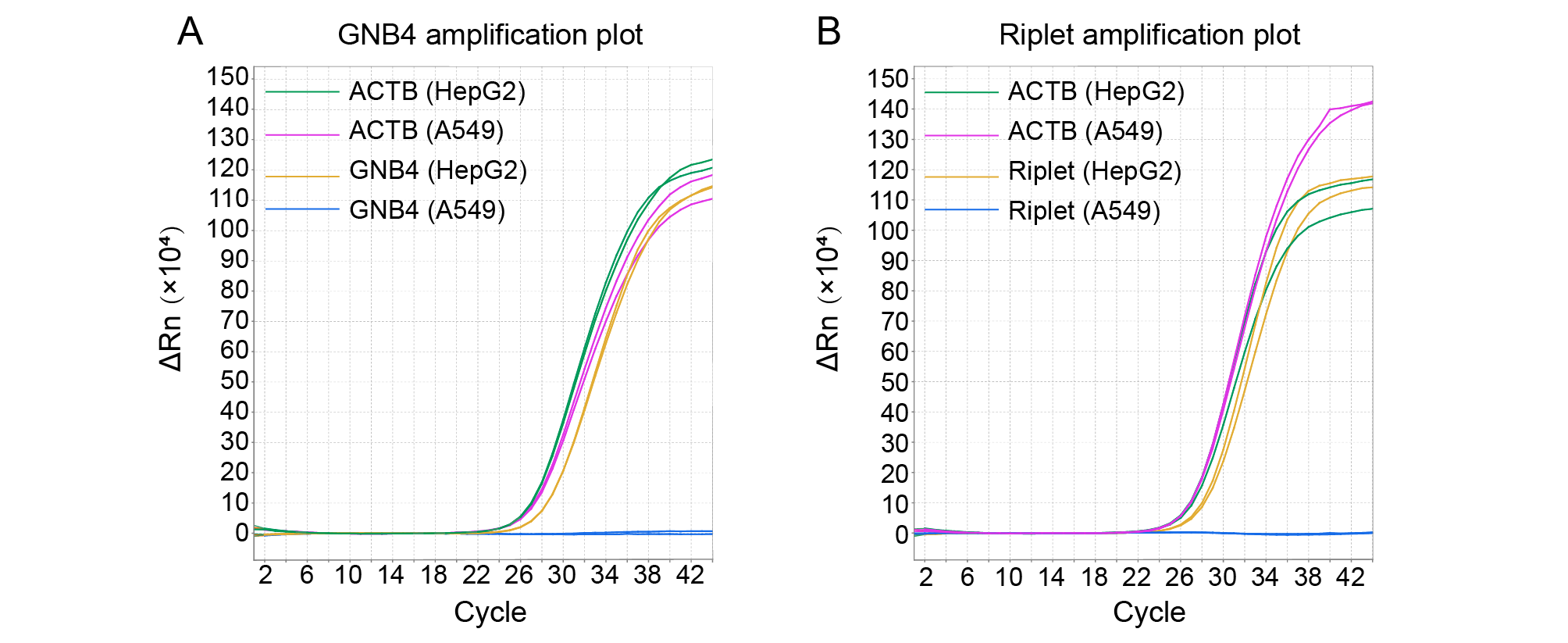


Supplemental Figure S2: Amplification curves of each primer in negative and positive cell lines.


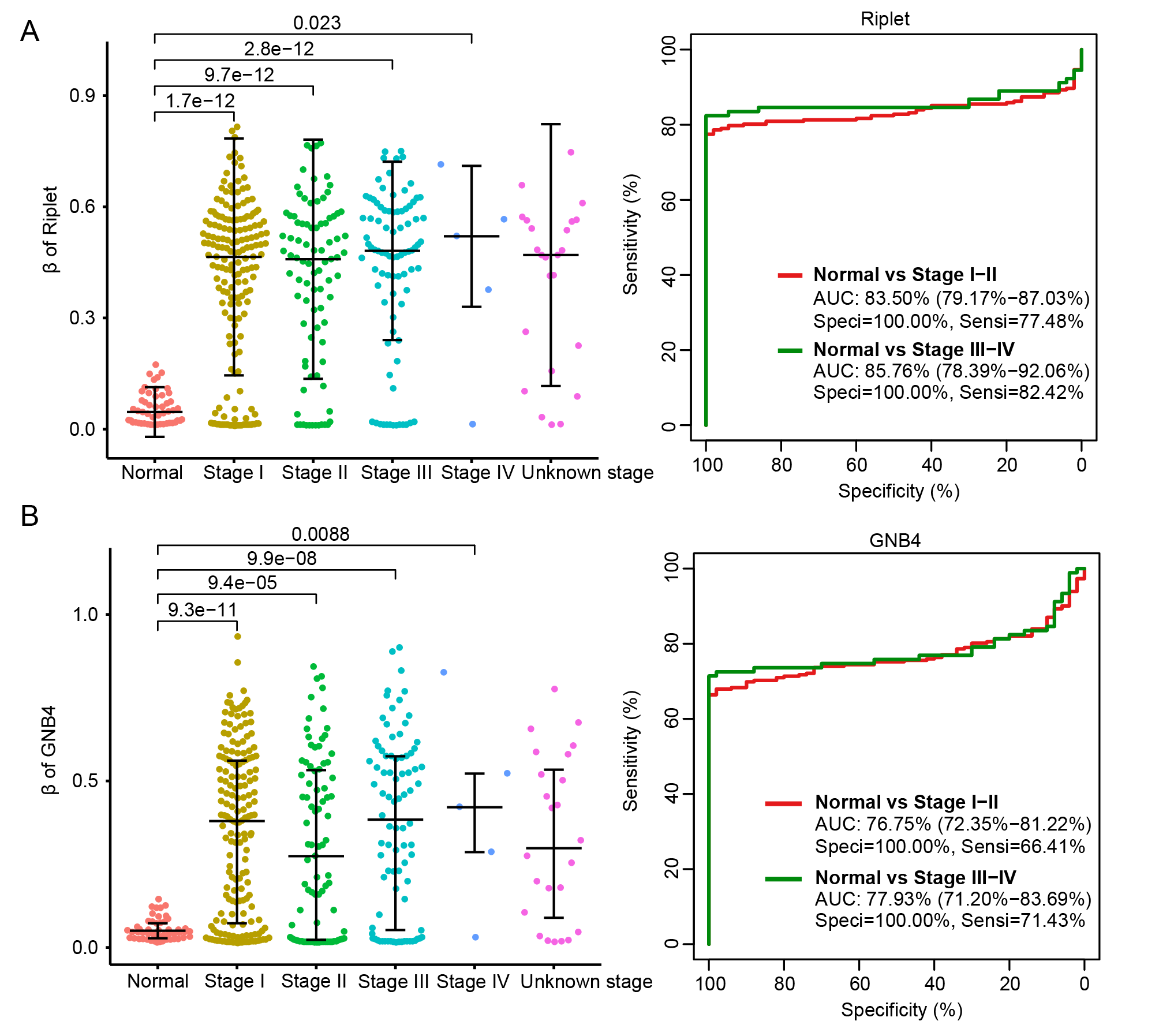


Supplemental Figure S3: The ROC analysis of the diagnostic performance of Riplet and GNB4 genes for different stages of HCC in TCGA database. A: The methylation levels (left) and ROC analysis (right) of Riplet gene in TCGA database; B: The methylation levels (left) and ROC analysis (right) of GNB4 gene in TCGA database.
